# CircHIPK3: Key Player in Pathophysiology and Potential Diagnostic and Therapeutic Tool

**DOI:** 10.3389/fmed.2021.615417

**Published:** 2021-02-22

**Authors:** Jiang Zhou, Baisheng Wang, Xin Bin, Changqing Xie, Bo Li, Ousheng Liu, Zhangui Tang

**Affiliations:** Hunan Key Laboratory of Oral Health Research & Hunan 3D Printing Engineering Research Center of Oral Care & Hunan Clinical Research Center of Oral Major Diseases and Oral Health & Xiangya Stomatological Hospital & Xiangya School of Stomatology, Central South University, Changsha, China

**Keywords:** CircHIPK3, miRNA, fibrosis, cancer, pathophysiological, treatment

## Abstract

A large number of studies in China and other countries have confirmed that circularHIPK3 (circHIPK3) plays an important role in the pathophysiological processes of various diseases. Through the action of sponge miRNA (miR), circHIPK3 regulates cell proliferation, differentiation, and migration, and plays a key role in disease processes. By referring to a large number of research reports, this article explores the specific functional role of circHIPK3 in fibrotic diseases, cancer, and other diseases. This review aims to clarify the role of circHIPK3 in disease processes in order to aid further studies into the specific pathogenesis and clinical diagnosis of various diseases and provide new ideas for treatments.

## Introduction

In 1976, Sanger et al. proposed the viroid to be a single-stranded covalently closed circRNA molecule and was the first human discovery of circRNA, a new type of non-coding endogenous RNA molecule. CircRNA, which exists in all eukaryotic cells, is formed by exon or intron cyclization, which connects the 3′ and 5′ ends to complete the circular structure. In the early stages of its discovery, circRNA was not generally recognized as being important because of the limitations of the traditional research methods (1–3). With the development of bioinformatics, research on circRNA is becoming increasingly in-depth. Numerous studies have shown that circRNA plays an important role in the pathophysiological processes of various diseases, such as cancer and cardiovascular disease, and in the development of neurons. Furthermore, studies have shown that circRNA also affects the occurrence and development of fibrotic diseases of organs such as the heart, lungs, and kidneys, playing a key role in organ fibrosis (1, 4–9). It has been shown that a member of the circRNA family, circHIPK3, is abnormally upregulated in organ fibrosis, cancer, and other diseases, suggesting that circHIPK3 is involved in these diseases. This review will primarily discuss the function of circHIPK3 in these conditions and the current relevant research progress and future prospects to provide new ideas for exploring the circHIPK3-related pathogenesis, clinical diagnosis, and treatment of cancer, fibrosis, and other diseases.

## CircRNA Biogenesis

**Figure 1:**
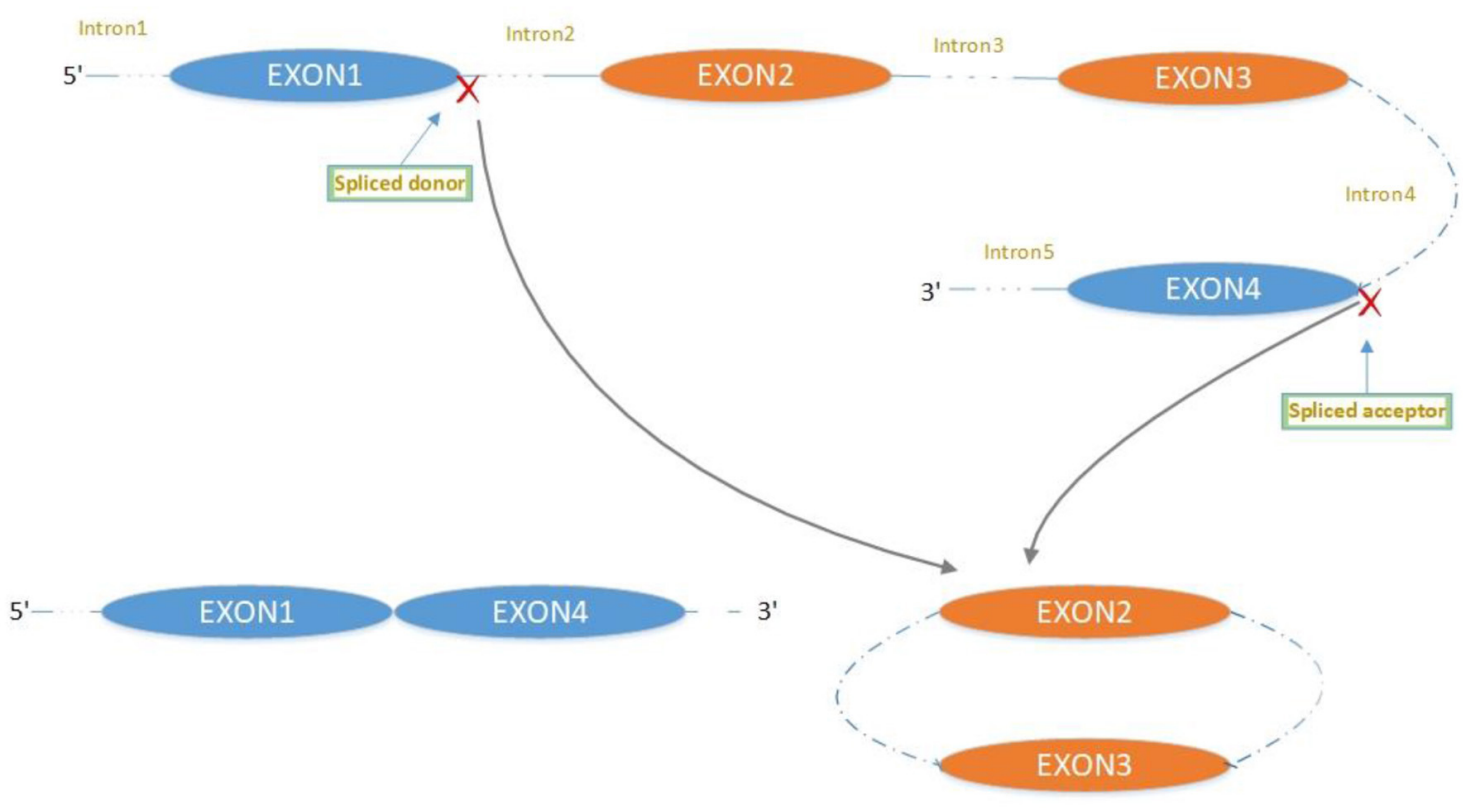
Mechanism of exon circularization to form circRNA: lasso-driven circularization.

**Figure 2:**
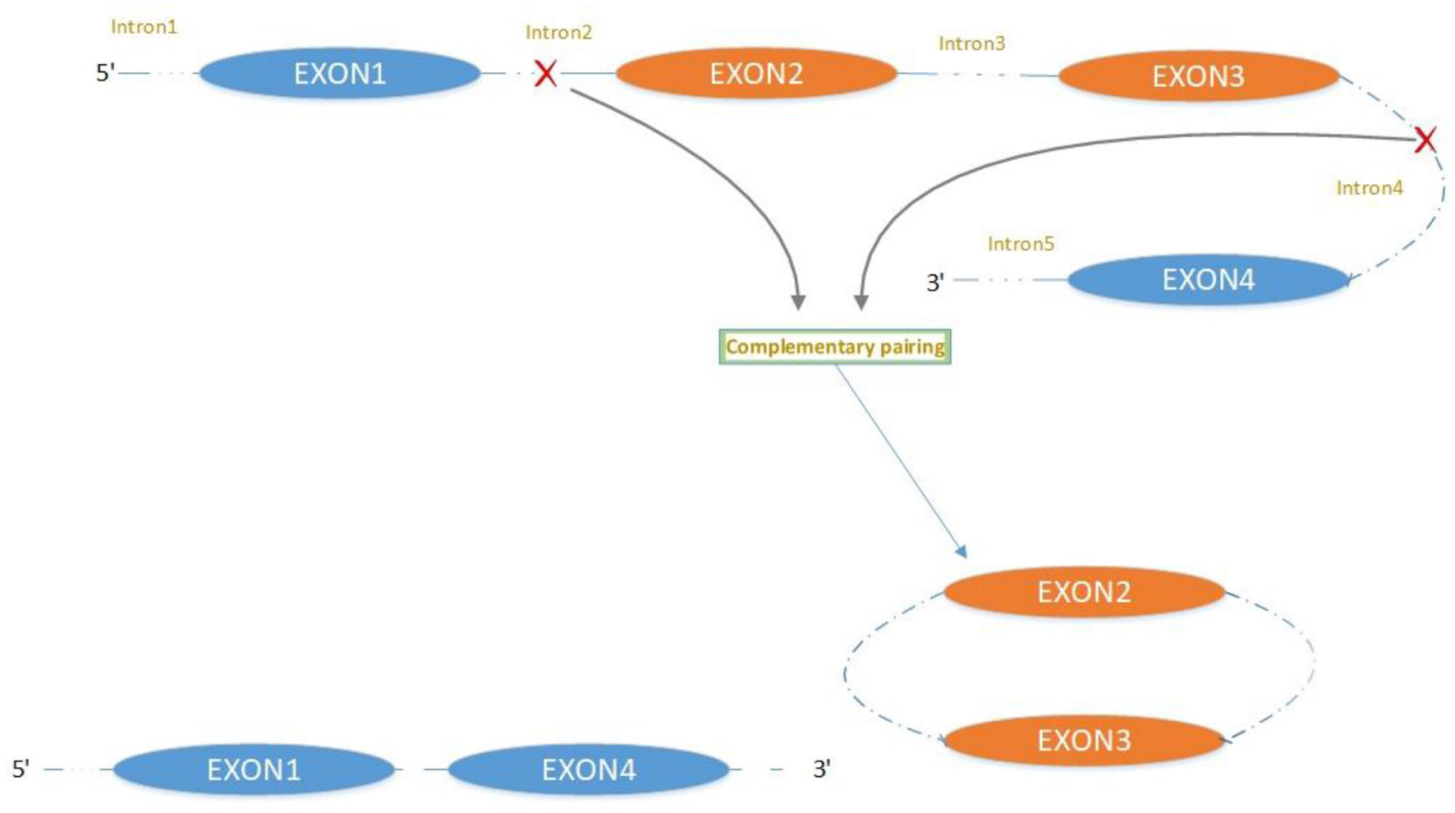
Mechanism of exon circularization to form circRNA: intron-pairing-driven circularization.

**Figure 3:**
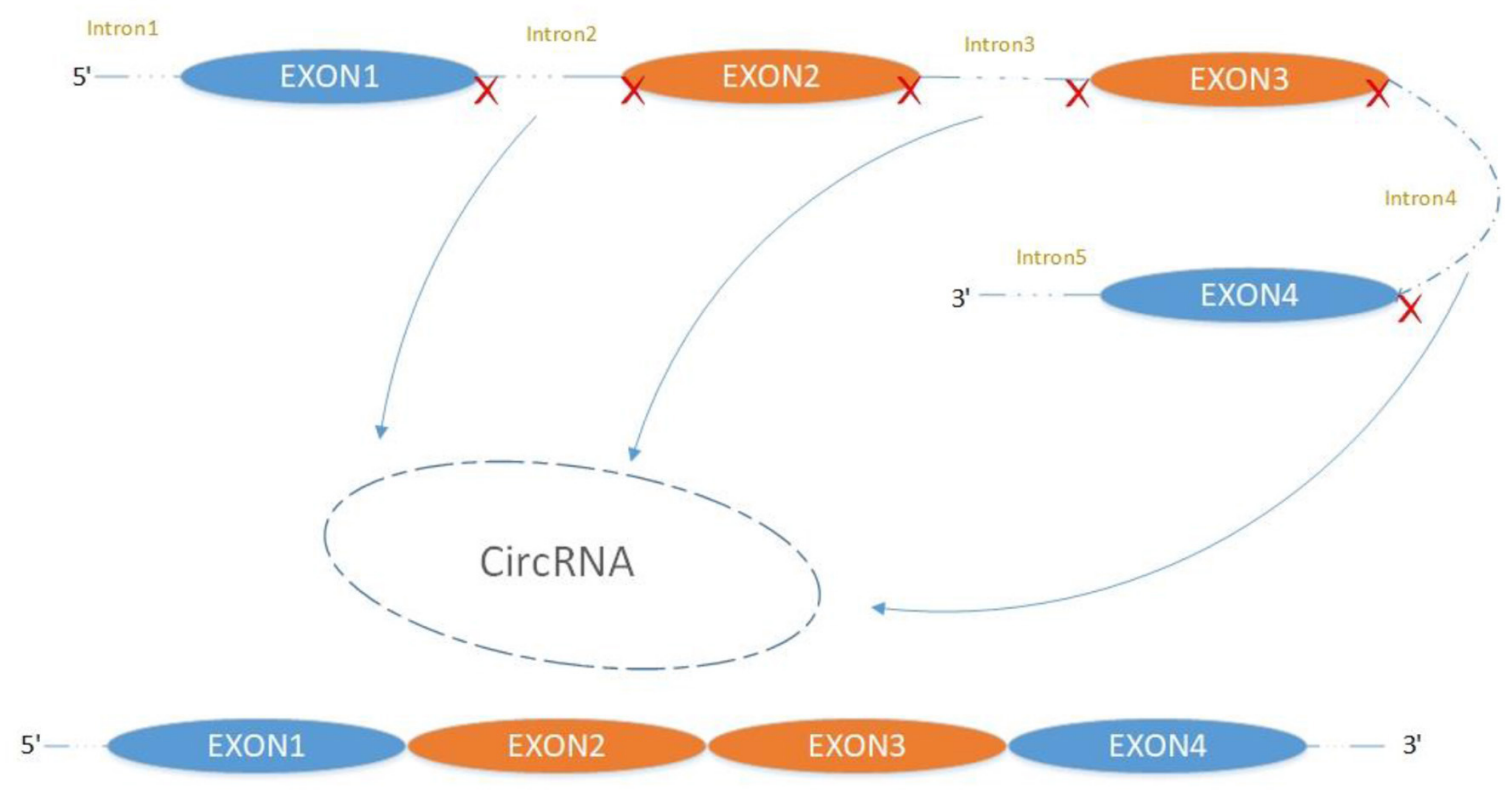
Mechanism of intron circularization to form circRNA.

The majority of circRNAs are produced by complete protein-coding gene exons. CircRNA undergoes circularization in two ways: lasso-protein-driven or intron-pairing-driven circularization. The former method of circularization involves exon skipping, i.e., the 3′ splice donor of one exon is covalently bound to the 5′ splice acceptor of the other exon—the lasso (10, 11). Intron-pairing-driven circularization starts with the codons of a splice donor and a splice acceptor, which are exonerated (Figure 1). After the circular structure is formed, the introns are removed to form circRNA (Figure 2). Studies have also confirmed that circRNA can form from intron circularization (Figure 3) (3–6). Because of the lack of a free 3′ or 5′ end, circRNA has a long half-life and is more stable than linear RNA. In terms of gene regulation, circRNAs have multiple functions: they (1) act as microRNA sponges that can competitively bind to miRNA; (2) regulate splicing or transcription; (3) interact with RNA-binding proteins; (4) are involved in translation; (5) regulate epigenetic alterations; and (6) transport substances and information (12, 13). Studies have shown that, because of the high production and accumulation rate, nervous tissue is rich in circRNA. In addition, circRNA is highly enriched in synapses, and its levels are regulated by neuronal activity, suggesting that circRNA has a specific role in the brain (2, 14, 15), and the latest research has shown this in the mammalian brain. Some circRNAs control gene expression by regulating mRNA production from host genes. Other circRNAs have trans-functionality, for example, CDR1 and Sry bind to specific miRNA as well as acting as miRNA sponges and/or transporters, and circ-muscleblind (circMbl) may bind the multifunctional protein muscleblind (MBL) (16, 17). Furthermore, other circRNAs are thought to bind to transcription factors, participate in muscle development or viral transcription, or have other functions.

## CircHIPK3 Biogenesis

The circularization of the second exon of the homology domain-interacting protein kinase 3 (HIPK3) gene produces circHIPK3, which is abundantly expressed in the cytoplasm of several human tissues including the lungs, heart, stomach, colon, brain, and other organs. The content of HIPK3 in the human body is particularly abundant, and the reverse splicing rate is high. HIPK3 is located on chromosome 11p13 and consists of 7,551 base pairs. Long flanking introns with complementary Alu repeat sequences are necessary for the formation of circHIPK3. Using luciferase experiments, Zheng et al. found that circHIPK3 has nine direct miRNA binding sites and 18 potential binding sites, and they concluded that circHIPK3 participates in mediating cell growth, proliferation, and migration through the sponging mechanism and plays an important role in the occurrence and development of various diseases (18–20). A large number of reports show that circHIPK3 plays a key role in the occurrence and development of fibrosis and tumors.

### The Role of circHIPK3 in Fibrosis Diseases

#### Cardiac Fibrosis

Cardiac fibrosis, which is a key feature of the pathological processes of cardiovascular disease, is due to the deposition of a large amount of extracellular matrix in the heart interstitium. This leads to the contraction and relaxation of the heart, ultimately leading to a series of heart conditions, including heart failure (21). Ni et al. found that, compared with normal heart tissue, circHIPK3 is expressed at a high level in cardiac fibrotic tissue and is induced by angiotensin II. Silencing circHIPK3 can inhibit the proliferation and migration of cardiac fibroblasts, but overexpression of circHIPK3 can promote the proliferation and migration of cardiac fibroblasts; circHIPK3 regulates the proliferation and migration of cardiac fibroblasts through the circHIPK3-miR-29b-3p pathway. A combination of circHIPK3 silencing and miR-29b-3p overexpression had a better inhibitory effect on the proliferation and migration of cardiac fibroblasts (22). Si et al. found that circHIPK3 is upregulated in the hearts of fetuses and newborns, and it is necessary for neonatal cardiomyocyte proliferation. In *in vitro* experiments, the overexpression of circHIPK3 promoted the proliferation and migration of cardiomyocytes. Furthermore, the overexpression of circHIPK3 promoted heart regeneration in adult mice. The circHipk3-miR-133a (the circHipk3-miR-133a-connective tissue growth factor axis) pathway in the myocardium participates in mediating the function and angiogenesis of human coronary artery endothelial cells (HCAECs) during infarction, while circHIPK3 induces cardiomyocyte proliferation by increasing the stability of notch1 intracellular domain (N1ICD) (23). Bai et al. found that circHIPK3 promotes myocardial ischemia/reperfusion injury by sponge miR-124-3p (24). In experiments using mice with myocardial infarction, Deng et al. found that circHIPK3 downregulation can reduce cardiac fibrosis and maintain cardiac function (25). Whether studying cardiac fibrosis or other cardiovascular diseases, scholars have found that circHIPK3 binds miRNA through the sponge mechanism to change the expression of the corresponding protein, thereby affecting the progression of the disease. These findings suggest that circHIPK3 plays an essential role in the process of cardiac fibrosis. However, these experiments were *in vitro* or animal experiments, so further studies are needed to verify this conclusion.

#### Pulmonary Fibrosis

Pulmonary fibrosis is a chronic, progressive, and fibrotic interstitial lung disease of unknown etiology that can lead to an irreversible decline in lung function, progressive respiratory failure, and even death. The specific pathogenesis of pulmonary fibrosis is still unclear. Recent studies have found that smoking and other factors lead to continuous alveolar epithelial cell damage and abnormal repair, the proliferation of fibroblasts, and the accumulation of extracellular matrix, leading to structural disorders of the lungs and, finally, fibrosis (26, 27). A study by Zhang et al. found that, in the bleomycin-induced pulmonary fibrosis mouse model, the expression level of circHIPK3 was upregulated in lung fibroblast-derived myofibroblasts.circHIPK3 participated in regulating the differentiation of fibroblasts into myofibroblasts and the proliferation of fibroblasts through the circHIPK3-miR338-3p/SOX4/COL1A1 pathway (28). Similarly to cardiac fibrosis, circHIPK3 increases the expression levels of SOX4 and COL1A1 by binding miRNA-338-3p in pulmonary fibrosis and thus promoting the progress of pulmonary fibrosis. The functional characteristics of circHIPK3 in fibrosis diseases are detailed in Table 1.

**Table 1:** The functional characteristics of CircHIPK3 in fibrotic diseases.

**Fibrotic diseases**	**CircHIPK3 expression level**	**Function**	**MiRNA**	**Related genes and proteins**	**Ref**.
Cardiac Fibrosis	Upregulated	Promoting Cardiac Fibrosis	MiR-29b-3p	α-SMA, COL1A1, COL3A3	(22)
Pulmonary Fibrosis	Upregulated	Promoting Pulmonary Fibrosis	MiR-338-3p	SOX4, COL1A1	(28)

*In cardiac fibrosis, circHIPK3 passes through sponge miRNA-29b-3p, leading to increased expression of α-SMA, COL1A1, and COL3A3, and thereby promoting the fibrotic process; In pulmonary fibrosis, circHIPK3 passes through sponge miRNA-338-3p, which leads to the increased expression of SOX4 and COL1A1, thereby promoting the progress of fibrosis*.

### The Role of circHIPK3 in Cancer/Tumoral Diseases

Numerous studies have shown that circHIPK3 is involved in the pathogenesis and prognosis of various cancers. Zeng et al. found that circHIPK3 promotes the growth and metastasis of colorectal cancer through sponge miR-7 (29), and Zhang et al. found that colorectal cancer patients resistant to oxaliplatin and those with colorectal cancer recurrence showed high levels of circHIPK3 expression and that circHIPK3, colorectal tumor size, regional lymph node metastasis, distant metastasis, and prognosis were closely related (30). In patients with chronic myeloid leukemia, circHIPK3 was found at high expression levels, suggesting that circHIPK3 is closely related to the prognosis of the disease (31). When Fang Teng et al. silenced circHIPK3, the proliferation, migration, and invasion of ovarian cancer cells and normal ovarian epithelial cells were promoted, and apoptosis was inhibited. Gao Wenzhe et al. found that the abnormal expression of circHIPK3 may be closely related to the prognosis of patients with malignant tumors (32).

Compared with adjacent tissues, the expression level of circHIPK3 was higher in gastric cancer cells compared with gastric mucosal cells, and the expression level of circHIPK3 was negatively correlated with the overall prognosis of gastric cancer patients. Silencing circHIPK3 weakened the proliferation and migration ability of gastric cancer cells. In addition, knocking out circHIPK3 significantly downregulated the levels of WNT1, TCF4, and β-catenin (33). Moreover, circHIPK3 was found to participate in the regulation of brain-derived neurotrophic factors by sponge miR-107, and it plays a further key role in the development of gastric cancer (34).

In another study, circHIPK3 promoted glioma progression by regulating the miR-654/IGF2BP3 signaling pathway and could be used as a prognostic indicator of glioma (35). Additionally, circHIPK3 was found to promote the proliferation and metastasis of gliomas through sponge miR-124-3p (36). Yawei Li et al. showed that sponge miR-558 and circHIPK3 inhibited the expression of heparinase in bladder cancer cells (37). The overexpression of circHIPK3 was found to promote the proliferation and metastasis of prostate cancer cells through sponge miR-338-3p (38); however, Dong Chen et al. demonstrated that the circHIPK3-miR-193a-3p-MCL1 signaling axis mediates the occurrence and development of prostate cancer (39).

Bioinformatics analysis has revealed the circHIPK3-miRNA-mRNA axis to be a potential signal pathway involved in the effects of circHIPK3 (40). In one study, circHIPK3 promoted the proliferation of human oral squamous cell carcinoma cells through sponge miR-124 (41). Whereas, in another study, circHIPK3 inhibited the progression of lung cancer via sponge miR-124 (42). In addition, circHIPK3 was shown to regulate the expression level of IGF1 through miR-379 to promote the proliferation of non-small cell lung cancer cell lines, NCI-H1299 and NCI-H2170 (43). According to Jiale Wang et al., circHIPK3 promoted acinar cell carcinoma apoptosis by regulating the miR-193a-5p/GSDMD pathway (41, 44). Jinjin Lai et al. showed that circHIPK3 promotes the proliferation and metastasis of renal cancer cells by downregulating miR-485-3p (45). The metastasis of osteosarcoma cells is mediated through the circHIPK3-miR-637/STAT3 signaling pathway (46, 47). In animal experiments, circHIPK3 promoted the proliferation and metastasis of esophageal squamous cell carcinoma cells by mediating the circHIPK3-miR-599/c-MYC signaling pathway (48).

High expression levels of circHIPK3 in tumor patients indicate that the tumor is at a relatively advanced stage. In addition, in the prognostic follow-up of patients with high levels of circHIPK3 expression, the probability of distant tumor metastasis and lymph node metastasis was higher than for other patients. Thus, the expression level of circHIPK3 plays an important role in the progression and prognosis of tumor diseases, suggesting that circHIPK3 may be a target gene for the diagnosis and treatment of related tumors. The functional characteristics of circHIPK3 in cancer are detailed in Table 2 (refer to Wen J, et al. Circular RNA HIPK3: A Key Circular RNA in a Variety of Human Cancers. Front Oncol. 2020 May 15;10:773, and supplement) (64).

**Table 2:** The functional characteristics of CircHIPK3 in cancer.

**Cancer types**	**Expression Roles**	**Functional**	**MiRNA**	**Related genes and protein**	**Ref**.
Lung cancer	Upregulated Tumor promoter	Viability, proliferation, apoptosis	MiR-124	SphK1, STAT3, CDK4	(42)
	Upregulated Tumor promoter	Proliferation, migration, autophagy	MiR-124-3p	STAT3	(49)
	N/A Tumor promoter	Proliferation	MiR-379	IGF1	(43)
	Upregulated Tumor promoter	Proliferation, migration, invasion, apoptosis	MiR-149	FOXM1	(50)
Gastric cancer	Upregulated Tumor promoter	Proliferation	MiR-124/MiR-29b	COL1A1, COL4A1, CDK6	(51)
	Downregulated Tumor suppressor	N/A	N/A	N/A	(52)
	Upregulated Tumor promoter	Proliferation, migration	N/A	WNT1, TCF4, β-catenin	(33)
Colorectal cancer	Upregulated Tumor promoter	Proliferation, migration, invasion, apoptosis, metastasis	MiR-7	c-Myb, FAK, IGF1R, EGFR, YY1	(29)
	Upregulated Tumor promoter	Proliferation, migration, invasion	MiR-1207-5p	FMNL2	(53)
Bladder cancer	Downregulated Tumor suppressor	Migration, invasion, angiogenesis, metastasis	MiR-558	HPSE	(37)
Nasopharyngeal carcinoma	Upregulated Tumor promoter	Proliferation, migration, invasion	MiR-4288	ELF3	(54)
Gallbladder cancer	Upregulated Tumor promoter	Viability, proliferation, apoptosis	MiR-124	ROCK1, CDK6	(55)
Hepatocellular carcinoma	Upregulated Tumor promoter	Proliferation, migration	MiR-124	AQP3	(56)
Osteosarcoma	Downregulated Tumor suppressor	Proliferation, migration, invasion	N/A	N/A	(57)
	Upregulated Tumor promoter	Proliferation, migration, invasion	MiR-637	STAT3	(46)
Glioma	Upregulated Tumor promoter	Proliferation, migration, invasion	MiR-654	IGF2BP3	(35)
	Upregulated Tumor promoter	Proliferation, migration, invasion	MiR-124-3p	WEE1	(36)
	Upregulated Tumor promoter	Proliferation, migration	MiR-124-3p	STAT3	(58)
Epithelial ovarian cancer	Upregulated Tumor promoter	N/A	N/A	N/A	(59)
Prostate cancer	Upregulated Tumor promoter	Proliferation, invasion	MiR-193a-3p	MCL1	(39)
Oral squamous cell carcinoma	Upregulated Tumor promoter	Proliferation	MiR-124	N/A	(41)
Chronic myeloid	Upregulated Tumor promoter	N/A	N/A	N/A	(31)
Leukemia	Upregulated Tumor promoter	Proliferation, migration, invasion	MiR-330-5p	RASSF1	(60)
Pancreatic cancer	Upregulated Tumor promoter	Proliferation, migration, invasion	MiR-485-3p	C caspase-3, Bax, Bcl-2, E-Cad, N-Cad, Vimentin	(45)
Clear Cell Renal Cell Carcinoma	Upregulated Tumor promoter	Proliferation, migration,	MiR-508-3p	CXCL13	(61)
Breast cancer	Upregulated Tumor promoter	Proliferation, migration, invasion	MiR-193a	HMGB1/PI3K/AKT	(47)
Esophageal Squamous Cell Carcinoma	Upregulated Tumor promoter	Proliferation, migration,	MiR-599	c-MYC	(48)
Cervical Cancer	Upregulated Tumor promoter	Proliferation, migration, invasion	MiR-338-5p	HIF-1α	(62)
Melanoma	Upregulated Tumor promoter	Proliferation, migration,	MiR-215-5p	YY1	(63)

*In most malignant tumor tissues, circHIPK3 shows high levels of expression. CircHIPK3 regulates the expression of the corresponding protein through sponging miRNA, promoting the occurrence and development of tumors. However, some malignant tumor tissues, such as gastric cancer, bladder cancer, and osteosarcoma, have been shown to have low levels of circHIPK3 expression. Therefore, circHIPK3 may have specific expression. In short, circHIPK3 plays a role as a cancer-promoting factor in most tumor diseases and regulates tumor cell functions by binding to the corresponding miRNA through the sponge mechanism*.

In short, these lines of evidence indicate that circHIPK3 is closely related to the progression and prognosis of various malignant tumors. In *in vitro* and *in vivo* experiments, the silencing and overexpression of circHIPK3 had significant effects on the proliferation, migration, and invasion of tumor cells, indicating that expression levels of circHIPK3 are closely related to the biological behavior of tumor cells. However, the recent experiments have had many limitations. To explore and further demonstrate the specific mechanisms of circHIPK3 in human diseases, additional experiments are needed.

### The Role of circHIPK3 in Other Diseases

CircHIPK3 is involved in the pathophysiological process of other diseases, including fibrotic diseases, cancer, and other diseases. Research found circHIPK3 promoted the expression of vascular endothelial growth factor C, FZD4, and WNT2 by sponge miR-30a-3p and, consequently, mediated diabetic retinal vascular dysfunction (65). High blood glucose levels led to downregulated circHIPK3 expression, promoting damage to vascular endothelial cells, while the overexpression of circHIPK3 inhibited vascular endothelial cell damage (66). Other studies have found that circHIPK3 is involved in the pathophysiological processes of diseases such as diabetic neuropathic pain, type 2 diabetes, diabetic nephropathy, acute pancreatitis, osteoarthritis, myocardial ischemia reperfusion injury, and age-related cataract, and plays an important role in these conditions (24, 44, 67–70). Although circHIPK3 is downregulated in age-related cataracts, and it is upregulated in all other fibrotic diseases. It also regulates cell proliferation, differentiation, and metastasis through the sponging mechanism, which affects the progression of diseases. The functional characteristics of circHIPK3 in other diseases are detailed in Table 3.

**Table 3:** The functional characteristics of CircHIPK3 in non-cancer diseases.

**Type of disease**	**CircHIPK3 expression level**	**Function**	**MiRNA**	**Related genes and proteins**	**Ref**.
Diabetic neuropathic pain/Type 2 diabetes	Upregulated	Promoting pain	MiR-124	IL-1β, IL-6, IL-12, TNF-α	(70)
Myocardial ischemia-reperfusion injury	Upregulated	Aggravating injury	MiRNA-124-3p	N/A	(24)
Acute pancreatitis	Upregulated	Promoting apoptosis	MiR-193a-5p	GSDMD	(44)
Diabetic nephropathy	Upregulated	Proliferation	MiR-185	N/A	(69)
Osteoarthritis	Upregulated	Promoting apoptosis	MiR-124	SOX8	(68)
Age-related cataract	Down-regulated	Promoting apoptosis	MiR-193a	CRYAA	(67)

*In addition to its downregulation in age-related cataracts, circHIPK3 is upregulated in myocardial ischemia-reperfusion injury, acute pancreatitis, diabetic nephropathy, osteoarthritis, and other diseases. CircHIPK3 also promotes the occurrence and development of diseases through sponge miRNA*.

## Conclusions and Future Prospects

CircRNA, a type of non-coding RNA, can regulate gene expression through a variety of mechanisms and may also have roles in regulating protein expression. The function of circRNA is complex, and its regulatory mechanisms are not fully understood. Current research has focused on changes in circRNA expression during physiological and pathological processes, and circRNA has become a new tool for understanding different diseases and their progression. Many scholars have been exploring the potential uses of the molecules in detecting disease states and for gene therapy. Functional research into circRNA in fibrotic diseases, cancer, and other diseases is a rapidly developing field, but these research avenues are still in the early stages and require follow-up efforts. CircHIPK3 is a member of the circRNA family. Numerous studies have shown that circHIPK3 is abnormally expressed in fibrotic diseases, cancer, and other diseases, which indicates that circHIPK3 may play an important role in pathogenesis. This review focused on the role of circHIPK3 in the pathophysiological processes of fibrotic diseases, cancer, and other diseases. As we have discussed, there is substantial information on the role of circHIPK3 in various diseases, which provide ideas for studying the pathogenesis of various diseases and clinical diagnosis and treatment of various diseases. Except for its downregulation in age-related cataracts, circHIPK3 is upregulated in all other diseases studied and can control cells through the sponging mechanism, affecting the cell cycle, cell proliferation, cell apoptosis, and invasion and migration. Furthermore, circHIPK3 can be used as a prognostic indicator of disease. Considering circHIPK3's unique stability, miRNA sponging function, and regulation ability, it may be a promising therapeutic drug carrier and a potential diagnosis and treatment target. We have discussed the role of circHIPK3 in heart and pulmonary fibrosis, but there are no reports on its role in oral submucosal fibrosis, and further studies are needed. Although there has been a plethora of research efforts to clarify the mechanisms of circHIPK3 action in various diseases, because of technological limitations, research has focused on the role of circHIPK3 as a microRNA sponge, and its other functions require further investigation. Based on the large number of studies that have shown correlation between circHIPK3 and various diseases, we have deepened our understanding of the role of circHIPK3 in pathogenesis. At the same time, it also provides a new way to explore the pathogenesis of different diseases. In addition, circHIPK3 has great clinical value in the diagnosis, treatment, and prognosis of fibrotic diseases, cancer, and other diseases. Although the specific mechanism through which circHIPK3 participates in various diseases is still unknown, we believe that, with the development of bioinformatics technology, the link between circHIPK3 and diseases will eventually be revealed.

## Author Contributions

JZ designed the manuscript, a major contributor in writing the manuscript, and drew the figures and tables. JZ, BW, XB, CX, and BL mainly revised the manuscript. JZ, OL, and ZT participated in the design of the review and made some revisions of the review. All the authors read and approved the final version of the review.

## Conflict of Interest

The authors declare that the research was conducted in the absence of any commercial or financial relationships that could be construed as a potential conflict of interest.
